# Research protocol: Cisplatin-associated ototoxicity amongst patients receiving cancer chemotherapy and the feasibility of an audiological monitoring program

**DOI:** 10.1186/s12905-017-0486-8

**Published:** 2017-12-11

**Authors:** J. Paken, C. D. Govender, V. Sewram

**Affiliations:** 1 Discipline of Audiology, School of Health Sciences, University of KwaZulu-Natal, Private Bag X54001, Durban, 4000 South Africa; 2 African Cancer Institute, Department of Global Health, Faculty of Medicine and Health Sciences, Stellenbosch University, P.O. Box 241, Cape Town, 8000 South Africa

**Keywords:** Cervical cancer, Cisplatin, Ototoxicity, Hearing loss, Quality of life, Audiology

## Abstract

**Background:**

Cisplatin is an anti-cancer chemotherapy drug classified as an alkylating agent. It is used for the treatment of a variety of cancers such as cervical, breast, stomach, prostate, bladder and oesophageal, to name a few. However due to its expansive toxicity profile, patients receiving cisplatin can experience high frequency hearing loss, a side effect known as ototoxicity. The dearth of information on the extent and severity of cisplatin-associated ototoxicity in South Africa prevents the implementation of a context-specific audiological monitoring programme.

**Methods:**

This study aims to determine the extent and severity of ototoxicity amongst patients with cervical cancer, receiving cisplatin-based chemotherapy and hence the feasibility of an ototoxicity monitoring program in the province of KwaZulu-Natal, South Africa. A concurrent mixed methods design will be employed in the study. This longitudinal study will involve interviewing oncology nurses, oncologists, pharmacists and audiologists to assess the level of awareness to ototoxicity, as well as conducting diagnostic audiological evaluations at regular intervals on 78 patients with cervical cancer to ascertain the progression of hearing loss during and after chemotherapy. The feasibility of the monitoring program will be assessed as a parallel process to the audiological evaluations, where patient outcomes and cost implications to the patient and the health sector will be considered. Data will be subjected to statistical analyses so as to strengthen knowledge in the field and inform appropriate policies, and healthcare providers.

**Discussion:**

This study is the first longitudinal study in South Africa to determine the ototoxic effects of cisplatin therapy on patients diagnosed with cervical cancer. Thus, the results generated from this study is likely to bring novel information to the fore using an evidence-based approach that will influence policy and clinical practice which can vastly improve the quality of life of patients undergoing chemotherapy. Mitigation of any further loss in the quality of life of affected patients is of paramount importance and the data generated from this project can lay the basis for further effective dialogue towards policy formulation on an ototoxic monitoring programme and the resultant strengthening of health systems in limited resource settings.

**Electronic supplementary material:**

The online version of this article (10.1186/s12905-017-0486-8) contains supplementary material, which is available to authorized users.

## Background

Ototoxicity refers to the hearing disorder which results from the temporary or permanent inner ear dysfunction after treatment with an ototoxic drug [1]. One such drug class that produces ototoxicity is the cancer chemotherapeutic agents. Chemotherapy is a core component of treatment for advanced cancers, when early metastasis is known to occur. As a result, a number of different cancer chemotherapy regimens are administered, depending on the type of cancer. As evidenced in Table 1 [2], a common thread is the use of cisplatin-based chemotherapy, as it is unique and unmatched in its effectiveness against many cancers [3].

**Table 1:** Types of cancers and the associated chemotherapy regimens [2]

Type of Cancer (incidence ranking)	Associated chemotherapy regimen
Males	
Basal cell carcinoma	Topical Fluorouracil
Prostate Cancer	Docetaxel and Prednisone
Squamous cell carcinoma	5-Flourouracil, Cisplatin or Carboplatin
Non-small cell lung cancer	Cisplatin, and Vinorelbine, or Docetaxel, or Gemcitabine, or Etoposide
Small cell lung cancer	Cisplatin or Carboplatin, Etoposide
Oesophageal Cancer	Cisplatin, 5-Fluorouracil, Docetaxel, Oxaliplatin, Capecitabine
Females	
Breast	Doxorubicin/Cycloplosphamide, Paclitaxel, Trastuzumab, Carboplatin, 5-Fluorouracil, Epirubicin
Cervix	Cisplatin, Paclitaxel, Carboplatin, Gemcitabine
Basal cell carcinoma	Topical Fluorouracil
Squamous cell carcinoma	5-Flourouracil, Cisplatin or Carboplatin
*Colon*	Oxaliplatin, Capecitabine, 5-Fluorouracil

However, while cisplatin chemotherapy is the treatment modality in advanced carcinogenesis, the resulting toxicity profile from the use of such regimens is expansive and affects the gastrointestinal, haematologic, renal and auditory systems [4].

In the auditory system, the primary site of cisplatin toxicity is the outer hair cells, with the basal turn of the cochlea appearing to be most affected [5]. However, if administration of cisplatin continues, damage to more apical areas is likely to occur [6]. Therefore, the initial manifestation of cisplatin-associated ototoxicity is the elevation of high frequency audiometric thresholds [7].

Cisplatin-associated ototoxicity usually manifests as irreversible, progressive, bilateral, high frequency sensorineural hearing loss associated with tinnitus [8]. The degree of hearing loss is often variable and is related to the dose. This is evident when one considers the findings of Bokemeyer et al. (1998) [9], who reported permanent hearing loss in 20% of patients who received cisplatin-based chemotherapy for testicular cancer. However, the incidence of hearing loss increased to 50% in another group of testicular cancer patients whose dosage was increased. An increased incidence of hearing loss in cancer patients receiving a higher dose of cisplatin was also reported by Waters et al. (1991) [6] and Dutta et al. (2005) [10].

While a review of the available literature revealed that there is only one study reporting on the incidence rates for cisplatin-associated ototoxicity in South Africa [11], a pooled analysis of various international studies indicated an overall incidence of about 62% [6]. This phenomenon is further supported by numerous studies highlighting the ototoxic nature of cisplatin in exposed patients. This is indicated by Waters et al. (1992) [6], who reported that 92% of their patients with ovarian cancer presented with cisplatin-associated ototoxicity. While investigating ototoxicity in patients with testicular cancer in a study conducted in German4y between 1977 and 1981, Strumberg et al. (2002) [12] reported that 7/30 patients presented with cisplatin-associated ototoxicity. This was in keeping with the findings of Bokemeyer et al. (1998) [9], who reported cisplatin ototoxicity in more than 50% of their cohort of 86 patients with testicular cancer in a study conducted in Hannover, Germany between 1976 and 1987. Similarly, Kopelman et al. (1988) [13] reported that 100% of their patients with advanced cancers showed some degree of hearing loss, while Nagy et al. (1999) [14] revealed that 36% of the 53 patients with oesophageal, lung or head and neck cancer presented with cisplatin-associated ototoxicity. Data from India also revealed similar findings [4, 10]. Whitehorn et al. (2014) [11] reported that 55.1% of the patients in their retrospective study developed ototoxicity while receiving high-dose (≥60 mg/m2) cisplatin treatment. Hence, it is evident that ototoxicity poses a major problem to the patient receiving cisplatin chemotherapy, as the quality of life during and after receiving such therapy can be negatively affected due to hearing loss resulting in social, emotional and vocational difficulties. Therefore, given these negative attributes, as a result of cisplatin, one has to ensure operational processes that will minimise the resulting co-morbidities from the use of such drug regimens.

An audiological monitoring program can avert, to a large extent, the reduced quality of life as a result of hearing loss, since patients on such drugs can be identified early, counselled, monitored and managed appropriately through interventions in a logical, systematic and coherent manner. However, there are currently no guidelines in South Africa for audiological management of ototoxicity. As a result, the “Guidelines for the audiological management of individuals receiving cochleotoxic drug therapy” developed by the American Association of Speech-Language-Hearing Association (ASHA) [15], and re-iterated in the “American Academy of Audiology Position Statement and guidelines: Ototoxicity monitoring” by the American Academy of Audiology(AAA) [16] is seen as the current gold standard and may, consequently, guide the Audiologist in the implementation of an ototoxicity monitoring program within a local, regional or national setting.

The ototoxicity monitoring protocol, proposed by ASHA [15] and AAA [16] represents an aggressive, ideal approach for monitoring ototoxicity, and is dependent on a country’s resources. It may, therefore, not always be feasible or suitable for a particular context. In addition, this protocol has been proposed by researchers in developed countries and has so far remained nothing more than an ideal for the South African Health care system, because no programmes have previously been formally implemented to identify and monitor ototoxicity in patients receiving cancer chemotherapy. As a result, there is no contextually relevant research to steer the implementation of an accountable and effective ototoxicity monitoring program in South Africa. The first step to developing and implementing such a program is to document the need within a specific context [17]. Therefore, knowledge of the epidemiology of hearing loss associated with cisplatin chemotherapy would form the basis for the implementation of such a program.

Studies in South Africa have focused on the awareness of healthcare personnel [18, 19] and reiterated the need for ototoxicity monitoring [1, 20]. In addition, whilst the prevalence of cisplatin ototoxicity was reported to be 55.1% in the Western Cape, South Africa, the study was retrospective in nature [11] and did not consider the use of previous ototoxic medication, which may have a cumulative effect on the hearing loss. The dearth of prospective data on the extent and severity of cisplatin-associated ototoxicity in South Africa hinders audiological clinical practice within the oncology context and thus the establishment of a contextually relevant ototoxicity monitoring programme.

As cisplatin-associated ototoxicity negatively affects quality of life, it is essential that a monitoring program, which identifies hearing loss early, is also implemented. In addition, it is necessary to determine the awareness of cisplatin-associated ototoxicity amongst health care personnel, as it has been reported to influence identification of patients at risk and hence early identification of hearing loss [18]. Moreover, a national programme of research and development would be important for “formulating integrated packages of care, clarifying steps to introducing them, testing how well such packages function, and establishing the cost and health gains from the integration of services” (p. 943) [21]. This, therefore, emphasizes the need to evaluate the feasibility of an audiological monitoring program within the South African context.

In light of the ototoxic nature of cisplatin and the dearth of information regarding its associated ototoxicity, this study seeks to evaluate hearing loss in a cohort of patients with cervical cancer. Cervical cancer, being the second most common cancer in South African females, has been selected due to the large number of patients receiving cisplatin chemotherapy at the recruitment facility. In addition, it was deemed appropriate to study the effects of cisplatin-associated ototoxicity in a relatively homogenous group (i.e. there is no variability in the number and frequency of treatments; type and dosage of other drugs administered in combination with cisplatin, type of cancer).

## Methods

### Aim

To evaluate the extent of cisplatin-associated ototoxicity in cervical cancer patients receiving chemotherapy and the feasibility of an audiological monitoring program.

### Objectives

2.2.1 To undertake a systematic review on the existence of ototoxicity induced by cancer chemotherapeutic regimens.

2.2.2 To determine the level of awareness of ototoxicity amongst health care personnel and current practices for monitoring ototoxicity.

2.2.3 To determine the extent and severity of ototoxicity amongst cervical cancer patients receiving cisplatin-based chemotherapy.

2.2.4 To implement, and evaluate a pilot monitoring program on ototoxicity with the feasibility of integration into the clinical environment.

### Study design

A concurrent mixed methods design will be employed in the study, with the aim of merging the qualitative and quantitative data in order to provide a complete analysis of the research problem [22]. The dominant paradigm is a **quantitative design**, i.e. panel study, which is to be conducted on patients diagnosed with cervical cancer and receiving cisplatin chemotherapy. Patient follow-up will be undertaken at regular intervals to ascertain the progression of the hearing loss, if any during and after cisplatin chemotherapy. The less dominant paradigm is the **qualitative design**, which will be used for the analysis of open-ended questions, and the subsequent evaluation of the feasibility of an audiological monitoring program through the use of the researcher’s field notes recorded during the implementation of the ototoxicity monitoring programme. Therefore, quantitative methodologies will be used for objectives 1, 2 and 3, while both quantitative and qualitative methodologies will be used for objective 4.

### Study population

The study will be conducted at Grey’s Hospital in the province of KwaZulu-Natal, South Africa. This is a referral hospital providing 20% regional and 80% tertiary services. It is also one of the main referral centres for cancer patients. In addition, the hospital also has an Audiology department; therefore, patients will not have to travel to other facilities for the audiological assessments.

The study population will comprise of:

1. Oncologists, nurses at the oncology clinic, pharmacists and audiologists employed at the hospital,
2. Patients diagnosed with cervical cancer and about to commence with cisplatin-based chemotherapy.

### Sampling strategy

Sampling would involve targeting all oncology clinic personnel, pharmacists, and audiologists as well as patients with cervical cancer at the hospital. There are currently 9 oncology nurses, 3 oncologists, and 4 medical doctors in the oncology department, 4 audiologists and 13 pharmacists at the site; therefore all will be targeted.

All patients with cervical cancer, commencing with cisplatin-based chemotherapy and meeting the inclusion criteria will be eligible for the study.

*Inclusion Criteria*

- Adults i.e. ≥18 years of age.
- Positive diagnosis of cervical cancer,
- Commencing with the first cycle of chemotherapy.

*Exclusion Criteria*

- Patients presenting with profound hearing loss at baseline assessment (as it may not be possible to determine if a significant hearing loss, as described by ASHA [15] develops).
- Patients who have previously received cisplatin chemotherapy (as the previous chemotherapy treatment may also contribute to the current ototoxic hearing loss).
- History of medical conditions such as tuberculosis, and malaria (as the medications used in the treatment of these conditions are ototoxic and may, therefore, confound the results).
- History of brain metastases as this may result in neurological complications and may thus confound test results.

### Sample size

A sample size of 78 achieves 80% power to detect a moderate effect size (W) of 0.3 using a 2 degrees of freedom (i.e. 2 groups) Chi-Square Test with a significance level (α) of 0.05 [23]. All new patients will be screened until the sample size is achieved. In addition, since attrition is a disadvantage of a longitudinal study [24] the researcher will attempt to account for it, by including a large number of participants at the onset of the study. An interim analysis will be conducted once half the sample size is enrolled. Sample size calculations are as follows:

**Table:** 

**F tests -** ANOVA: Repeated measures, between factors
**Analysis:** A priori: Compute required sample size
**Input:** Effect size f = 0.25 α err prob = 0.05 Power (1-β err prob) = 0.80 Number of groups = 2 Number of measurements = 5 Corr among rep measures = 0.5 **Output:** Noncentrality parameter λ = 8.125 Critical F = 3.967 Numerator df = 1.000 Denominator df = 76.000 **Total sample size = 78**

To account for attrition of 10%, the study intended to recruit 86 participants at the outset or until the desired sample size of 78 was achieved.

### Data collection

#### Objective 1: Systematic review

Data for the systematic review will be identified on Pubmed, Science Direct, Ebsco Host and Google Scholar for all medical research published in English-language journals up to December 2014 using the search terms “ototoxicity”, “cisplatin”, “chemotherapy” and “hearing loss”. Observational study designs (i.e. cross sectional and Cohort studies) will be accepted, provided that the intervention is cisplatin-based chemotherapy, the participants have been diagnosed with cancer and the outcome is hearing loss. The primary investigator and the supervisors will screen all the references to be included in the systematic review. If data is duplicated in more than one study, the most recent study will be included. A standardized reporting form will be used to abstract the following data from each publication: reference (first author, year of publication), study design, country in which study was performed, number of participants, variables examined, covariate adjustment, and methods used for ototoxicity monitoring.

#### Objective 2: Health care personnel’s knowledge of ototoxicity and current monitoring practices

Oncology clinic personnel i.e. oncologists and oncology nurses, will be provided with a self-administered questionnaire following informed consent. It is expected that the questionnaire will take approximately 10 min to complete. The questionnaire, adapted from de Andrade et al. (2009) (see Additional file 1), will include questions related to the following areas: clinical experience, management of patients on chemotherapy, ototoxicity and monitoring program.

Pharmacists will also be provided with a self-administered questionnaire (see Additional file 2) following informed consent. It is expected that the questionnaire will take approximately 10 min to complete. The questionnaire will include questions related to the following areas: experience, identification of patients at risk for hearing loss, ototoxicity and monitoring program.

The resident audiologists will be interviewed individually using a structured questionnaire (see Additional file 3). The interview will last approximately 30 min and will be audio recorded. The following general aspects will be covered during the interview: clinical experience, description of client base, ototoxicity and the ototoxicity monitoring program.

### Objective 3: Audiological assessment for ototoxicity

**Fig. 1:**
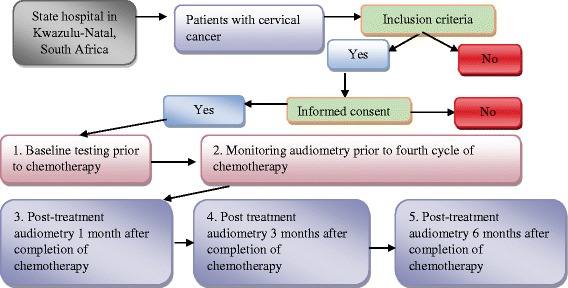
Data collection process for objective 3

Patients meeting the inclusion criteria will be invited to participate in the study. Following informed consent, patients will undergo audiological assessment through a battery of five tests prior to commencement of chemotherapy. These tests are routine procedures, considered non painful with minimum discomfort to the patient. Patients with cervical cancer undergo cisplatin chemotherapy cycles weekly for a maximum period of 6 weeks. Patient follow-up will, therefore, be conducted at the beginning of the fourth cycle and then at 1, 3 and 6 months after their last chemotherapy cycle (see Fig. 1). The monitoring audiometry will be conducted prior to the fourth cycle of chemotherapy, as this is generally the mid-point of the treatment regimen for the patient with cervical cancer. The 3-month follow-up audiological evaluation will be conducted as it would permit determining when an ototoxic hearing loss develops and would thus inform the ototoxicity monitoring programme. All study appointments will be coordinated using the oncology clinic register. However, patients will be encouraged to schedule an audiological evaluation between scheduled appointments, if they experience any otologic symptoms.

Audiological testing for participants who are responsive or whose responses are limited, would involve the following procedures:

- Review of patient’s medical records (for confirmation of diagnosis and medication),
- interview,
- otoscopic examination,
- immittance audiometry (tympanometry and acoustic reflex threshold testing),
- pure tone audiometry (air conduction and bone conduction),
- speech audiometry,
- Distortion product otoacoustic emission testing (DPOAEs) and
- Counselling and referral to Otolaryngologist (if significant changes in threshold identified).

Audiological testing for participants who are non-responsive would involve the following procedures:

- Review of patient’s medical records (for confirmation of diagnosis and medication),
- otoscopic examination,
- immittance audiometry (tympanometry and acoustic reflex threshold testing),
- DPOAEs and,
- Counselling and referral to Otolaryngologist (if significant changes in threshold identified). Therefore, only objective audiological tests will be used.

Prior to the commencement of each audiological procedure, appropriate instructions will be provided to the participants. The entire battery of audiological tests is estimated to take approximately 45 min. Patients expressing fatigue will be afforded short breaks before continuation. The audiological procedures indicated for ototoxicity monitoring will be utilized [15, 25] (see Additional file 4). The case history questionnaire, used in the study appears in Additional file 5, while all audiological results will be recorded on an audiogram, designed by the researcher for the purpose of the study (see Additional file 6). Information on the risk factors for ototoxic hearing loss will be documented during the case history interview. This questionnaire will also include questions on tinnitus, and caters for information related to concurrent use of antioxidants or dietary supplements by asking the patient about the use of all other medication apart from cisplatin.

All audiometric test results will be determined according to the norms (see Additional file 7). On the identification of a significant hearing loss, an audiological retest will be conducted within 24 h to verify the change [15]. On identification of a reduction in the hearing ability, participants will be counselled regarding treatment options such as compensatory communication strategies, as well as rehabilitation technology options and referred to the necessary medical personnel. Participants will also be encouraged to stay away from noisy environments as it would exacerbate the hearing loss.

#### Objective 4: Monitoring program

Patients will be monitored upon recruitment using the six fundamental elements of ototoxicity monitoring i.e. audiometric criteria for cochleotoxicity, identification of patients, pre-treatment counselling regarding the potential effects of the treatment on the auditory system, baseline testing prior to treatment, monitoring tests at intervals suitable to enable the earliest detection of the hearing loss, and follow up tests at intervals suitable to determine post treatment hearing status.

The ASHA [15] criteria will be used to identify significant ototoxic hearing loss. Identification of patients at risk for ototoxic hearing loss will be facilitated by conducting an information session with oncology clinic personnel and pharmacists. The nurse personnel will identify and inform the researcher of patients requiring ototoxicity monitoring, and assist with tracking patients. The researcher, together with the nurse will coordinate test schedules with treatment. Pre-treatment counselling will be provided by the researcher. This would involve explaining the potential effects of the treatment on the auditory system, as well as the purpose, benefits, and procedures involved with ototoxicity monitoring. The next three components of the monitoring program i.e. the audiological evaluations would be conducted, as indicated for objective 3.

Apart from monitoring patient outcomes, the following parameters relating to feasibility will also be assessed as a parallel process: number of patients with cervical cancer on cisplatin chemotherapy, number of patients presenting with cisplatin-associated ototoxicity, cost implications with regard to personnel, equipment and the patient with cervical cancer as well as the benefit of such a programme. Data for this objective will be recorded on a tracking document together with field notes. Oncology clinic personnel and pharmacists will be required to complete a questionnaire (see Additional file 8), and the researcher’s field notes will also be used to determine the feasibility of the monitoring program. In addition, participants with cervical cancer will be required to complete a questionnaire post the monitoring program to determine patient satisfaction (see Additional file 9).

### Data analysis

A statistician from the University of KwaZulu-Natal will assist with the statistical analysis of data.

#### Objective 1: Systematic review

A chi-square test for heterogeneity will be undertaken for the included studies. If heterogeneity is found, then a sensitivity analysis will be undertaken to identify factors that can explain it. A funnel plot will be used to assess the potential for publication bias. Meta-analysis will be undertaken using a random effects model.

#### Objective 2: Health care personnel’s knowledge of ototoxicity and current monitoring practices

Thematic analysis will also be used and common themes will be highlighted and grouped together to establish major themes in the analysis of the interviews with the audiologists and the open ended questions of the questionnaires. Descriptive statistics will be used to analyze the data. The goal of the descriptive statistics will be to provide a summary measure of some characteristic of the sample data [26].

Descriptive analysis methods will be used in terms of percentage counts, bar graphs and pie charts to analyse the results obtained in the study. The awareness categories will be obtained using a Likert scale. The data will then converted into percentages and frequency counts to allow for interpretation. The following criteria were developed in order to evaluate the level of awareness of hearing loss induced by cancer chemotherapy amongst healthcare personnel. The researcher classified awareness into four levels, namely “poor”, “average”, “good” and “excellent”. The levels were chosen by splitting the range of awareness scores into four levels. The lowest range (0–25%) would represent “poor” awareness, then 26–50% would represent “average” awareness, while 51–75% would represent “good” awareness and 76–100% would represent “excellent” awareness. Typically, with a large enough sample size, the minimum awareness score would be 0% and the maximum awareness score would be 100%. However, with the small sample size used in this study, the minimum and maximum values may not be at 0% and 100% respectively, but rather will be calculated based on the participant response rate.

#### Objective 3: Audiological assessment for ototoxicity

Patient screening, enrolment and follow-up will be described using a flowchart and reasons for exclusions and loss to follow-up reported. Descriptive statistics will be used to summarize baseline demographic and clinical characteristics of the participants. Each audiological assessment (conventional audiometry, extended high frequency audiometry and DPOAEs) will be summarized at each time point (baseline[T0], beginning of fourth cycle [T1], at 1 month post treatment [T2], 3 month post treatment [T3], and 6 month post treatment [T4]). Frequency distributions of quantitative data will be examined for normality. If the assumption of normality is not satisfied, data will be transformed or non-parametric statistics used in the analysis. The degree of hearing loss will be categorized at each time point. Quantitative changes in hearing loss will be analysed using a mixed model with assessment (the dependent variable); patient and ear (random variables) and time (the independent variable). Covariates such as age and use of antiretroviral medication will be examined as possible confounders or effect modifiers through logistic regression modelling to adjust for such confounders. Severity of hearing loss measured on an ordinal scale at baseline will be compared to that at each time point using a Wilcoxon rank sum test. Hearing loss will be dichotomized and a proportional-odds model will be used to look at changes over time. This model takes into account the clustered nature of the data. A Kaplan Meier curve will be used to examine ototoxic change and difference between the audiology tests.

#### Objective 4: Monitoring programme

The feasibility of the ototoxicity monitoring programme will be assessed by determining the cost of the programme to the Department of Health, healthcare personnel and the patient with cervical cancer. The need for the ototoxicity programme will be determined by calculating the percentage of participants with ototoxic hearing loss. Costs will be projected by determining how many audiologists will be required to sustain such a programme. In addition, the costs of the audio booth and the necessary audiological equipment will be determined by acquiring quotes from various distributors.

Cost to the patient with cervical cancer will be determined by analysing the time taken for each audiological evaluation as well as if the audiological evaluation is conducted on the same day as the chemotherapy cycle or if the patient needs to return on a separate day for the test. Furthermore, it will be determined if patients will have to purchase their own hearing aids (if required) or if the cost will be incurred by the Department of Health. In addition, the cost to the participants with cervical cancer will be determined by analysing the participants’ responses to the questionnaire following the final audiological assessment. Further, data from this questionnaire will also be analysed to determine the benefit of the ototoxicity monitoring program, by determining if participants felt that they received adequate counselling and were given the appropriate support, if required.

In addition, the field notes will be subjected to systematic analysis according to the general themes of context (e.g. facilities, barriers and positive aspects), collaboration with health care personnel (attitudes, contact, involvement, etc.) and experiences with participants (attitudes, physical state, collaboration, insight, etc.) [27]. The questionnaires completed by the oncology clinic personnel, and pharmacists will be analysed using thematic analysis and common themes will be highlighted.

### Pilot study

The questionnaire for the clinic personnel, pharmacists, the case history questionnaire, for the patients on chemotherapy will be piloted on 2 oncology clinic personnel, 2 pharmacists and 5 patients with cancer from the hospital, to ensure its reproducibility, as these questionnaires are considered to be newly devised instruments. The questionnaires will be completed at two different times (2 weeks apart) and intra-class correlation coefficients will be calculated between the first and second measurements of selected variables. The intra-class correlation coefficients will be calculated using the Kappa statistic.

### Ethical and legal considerations

The study has adhered to the principles of the Declaration of Helsinki and has received ethical clearance from the Biomedical Research and Ethics Committee of University of KwaZulu Natal (BE 064/13) (see Additional file 10), KwaZulu-Natal Department of Health and Greys Hospital. The study has also been funded by the National Department of Health and administered by the Medical Research Council (see Additional file 11).

## Discussion

This study is the first prospective follow up study in South Africa to determine the ototoxic effects of cisplatin therapy on patients diagnosed with cervical cancer. Recruitment of participants will require the assistance of the staff at the oncology department i.e. doctors and nurses, who will inform participants of the nature and purpose of the study. This will be expedited through a workshop on cisplatin-associated ototoxicity that will be facilitated by the primary researcher. Given that this study will be undertaken in a limited resource setting, patients do not always keep to appointments due to the vast distances that have to be travelled. Therefore attrition was considered and accounted for in the sample size calculation. Further, the primary researcher will ensure there is telephonic contact with participants together with the use of Short Message System (SMS) text services on mobile phones reminding participants of appointments. This study is expected to yield impacts in the following ways:

1. A greater understanding of ototoxicity risk amongst patients receiving cisplatin chemotherapy;
2. Primary data to assess, evaluate and inform a context-specific ototoxicity monitoring program within a limited resource setting;
3. Greater awareness on the ototoxic effects of cisplatin-associated chemotherapy amongst health care providers and the practice of hearing conservation among affected patients.

This study is also expected to yield as outputs:

1. A Doctoral Thesis,
2. Peer-reviewed publications based on the results obtained, and
3. A policy brief on ototoxicity monitoring within the South African context.

## Additional files

Additional file 1: Interview questionnaire for clinic personnel (PDF 162 kb)

Additional file 2: Interview questionnaire for pharmacists (PDF 88 kb)

Additional file 3: Interview questionnaire for audiologists. (PDF 85 kb)

Additional file 4: Audiological procedures to be utilized for data collection for objective 3. (PDF 173 kb)

Additional file 5: Interview questionnaire for patients. (PDF 103 kb)

Additional file 6: Audiogram. (PDF 702 kb)

Additional file 7: Measurements and equipment for audiological evaluations. (PDF 177 kb)

Additional file 8: Interview Questionnaire for oncology clinic personnel and pharmacists post ototoxicity program. (PDF 158 kb)

Additional file 9: Questionnaire for participants with cancer post monitoring program. (PDF 154 kb)

Additional file10: Ethical Approval from University of Kwazulu-Natal. (PDF 24 kb)

Additional file 11: Funding approval from South African Medical Research Council. (PDF 292 kb)

Additional file 12: Information document for clinic personnel. (PDF 197 kb)

Additional file 13: Information document for patients. (PDF 308 kb)

Additional file 14: Consent document for clinic personnel. (PDF 196 kb)

Additional file 15: Consent document for patients. (PDF 196 kb)

## Abbreviations

AAA: American Academy of Audiology
ASHA: American Speech and Hearing Association
DPOAEs: Distortion Product Oto-acoustic Emission
