# Supplementary material for: Research protocol: Cisplatin-associated ototoxicity amongst patients receiving cancer chemotherapy and the feasibility of an audiological monitoring program
Source: BMC Womens Health. 2017 Dec 11;17:129. doi: 10.1186/s12905-017-0486-8 (PMC5725900; doi:10.1186/s12905-017-0486-8)
Supplement: Supplementary file 1 — Interview questionnaire for clinic personnel (PDF 162 kb) [file 12905_2017_486_MOESM1_ESM.pdf]

Cisplatin-associated ototoxicity amongst patients receiving cancer chemotherapy and the feasibility of an audiological monitoring program

INTERVIEW QUESTIONNAIRE FOR CLINIC PERSONNEL

Dear Participant

We are delighted that you have agreed to participate and would like to thank you sincerely, as the information from this study can be used to help us understand the complexities associated with chemotherapy. The information that you provide will be treated with the strictest of confidence and please do not hesitate to ask us any questions that you may have during the course of the study. Contact details are reflected on the information and consent document.

INSTRUCTIONS

1. Please mark the appropriate answer to each question with an X, and give further detail if necessary.

2. Please answer all questions.

1.1. Please indicate your profession.

Oncologist	Oncology nurse

1.2. How long have you been working with patients with cancer?

Years	Months

2.1. Do the patients, on cancer chemotherapy, complain of any auditory symptoms?

Yes	No

2.2. If so, which of the following auditory symptoms do patients complain of?

Reduced hearing sensitivity	Pain in the ears	Noises in the ears	Hypersensitivity to sounds

2.2.1. If a patient experiences reduced hearing sensitivity, who is s/he referred to?

Family doctor	ENT specialist	Speech language pathologist	Audiologist

2.3. Are you aware that certain chemotherapy drugs, for cancer, may cause hearing loss?

Yes	No

2.3.1. Do you give the patients information about the possible ototoxic effects of the medication prior to commencing chemotherapy?

Yes	No

2.3.2. Do you provide the patient with any recommendations regarding their hearing?

Yes	No

2.3.3. If so, what are these recommendations?

____

____

2.4. Do you consider the audiologist to be part of the team who deals with patients with cancer?

Yes	No

2.5. Whose responsibility is it to provide patients with information about the possible ototoxic effects of medication?

Nurses	Oncologists	Pharmacists	Audiologists

2.6. Do you enquire about patient's history of hearing difficulties? Yes No

2.7.Do you enquire about family history of hearing loss? Yes No

2.8. Do you ask about the patient's medical history and drugs used to treat these conditions?

Tuberculosis	HIV	Malaria	Pain and fever

3.1. Does the oncology department have an ototoxicity monitoring program?

Yes	No	Not sure

3.2. Is there a protocol in the oncology department that indicates when a patient's hearing should be monitored?

Yes	No	Not sure

3.3. If yes, when should the patient, with cancer, be referred for a hearing test?

____

____

____

------------------------THANK YOU FOR YOUR TIME AND CO-OPERATION---------------
