# Supplementary material for: Research protocol: Cisplatin-associated ototoxicity amongst patients receiving cancer chemotherapy and the feasibility of an audiological monitoring program
Source: BMC Womens Health. 2017 Dec 11;17:129. doi: 10.1186/s12905-017-0486-8 (PMC5725900; doi:10.1186/s12905-017-0486-8)
Supplement: Supplementary file 2 — Interview questionnaire for pharmacists (PDF 88 kb) [file 12905_2017_486_MOESM2_ESM.pdf]

**Cisplatin-associated ototoxicity amongst patients receiving cancer chemotherapy and the feasibility of an audiological monitoring program**

## INTERVIEW QUESTIONNAIRE FOR PHARMACISTS

Dear Pharmacists

We are delighted that you have agreed to participate and would like to thank you sincerely, as the information from this study can be used to help us understand the complexities associated with chemotherapy. The information that you provide will be treated with the strictest of confidence and please do not hesitate to ask us any questions that you may have during the course of the study. Contact details are reflected on the information and consent document.

INSTRUCTIONS

1. Please mark the appropriate answer to each question with an X, and give further detail if necessary.

2. Please answer all questions.

1. Do you dispense medication to patients on cancer chemotherapy? Yes No

2. How many times in the last month have you been consulted about drug interactions?

   <10 10-19 20-29 30-39 40-50

3. Does the hospital have a system that would allow you to identify patients at risk for a hearing loss? Yes No

4. Are you aware that certain chemotherapy drugs, for cancer, may cause hearing loss?

   Yes No

5. Do you alert oncology staff to patients who are at risk for an ototoxic hearing loss?

   Yes No

6. Do you consider the audiologist to be a part of the team who deals with patients with cancer? Yes No

7. Whose responsibility do you think it is to provide patients with information about the possible ototoxic effects of medication?

   Nurses Oncologists Pharmacists Audiologists

8. Whose responsibility do you think it is to refer patients at risk for a hearing loss to the ototoxicity monitoring program?

   Nurses Oncologists Pharmacists Audiologists

-------------------------**THANK YOU FOR YOUR TIME AND CO-OPERATION**----------------
