# Supplementary material for: Research protocol: Cisplatin-associated ototoxicity amongst patients receiving cancer chemotherapy and the feasibility of an audiological monitoring program
Source: BMC Womens Health. 2017 Dec 11;17:129. doi: 10.1186/s12905-017-0486-8 (PMC5725900; doi:10.1186/s12905-017-0486-8)
Supplement: Supplementary file 3 — Interview questionnaire for audiologists. (PDF 85 kb) [file 12905_2017_486_MOESM3_ESM.pdf]

Cisplatin-associated ototoxicity amongst patients receiving cancer chemotherapy and the feasibility of an audiological monitoring program

INTERVIEW QUESTIONNAIRE FOR AUDIOLOGISTS

Dear Audiologist

We are delighted that you have agreed to participate and would like to thank you sincerely, as the information from this study can be used to help us understand the complexities associated with chemotherapy. The information that you provide will be treated with the strictest of confidence and please do not hesitate to ask us any questions that you may have during the course of the study. Contact details are reflected on the information and consent document.

INSTRUCTIONS

1. Please mark the appropriate answer to each question with an X, and give further detail if necessary.

2. Please answer all questions.

1. How many years have you been practicing Audiology?

____

2. Describe your client base.

____

____

3. Are aware of drugs that may result in hearing loss? ☐ Yes ☐ No

3.1. If yes, list some of the classes of drugs that may lead to this problem.

____

____

4. Describe the auditory complaints that patients on chemotherapy may complain of.

____

____

5. Discuss your role as part of the team who deals with patients with cancer.

____

____

6. What do you think are the key elements of an ototoxicity monitoring program?

____

____

7. Do you see a role for Audiologists in the above program? ☐ Yes ☐ No

    7.1. If yes, discuss your role.

____

____
