# Supplementary material for: Research protocol: Cisplatin-associated ototoxicity amongst patients receiving cancer chemotherapy and the feasibility of an audiological monitoring program
Source: BMC Womens Health. 2017 Dec 11;17:129. doi: 10.1186/s12905-017-0486-8 (PMC5725900; doi:10.1186/s12905-017-0486-8)
Supplement: Supplementary file 4 — Audiological procedures to be utilized for data collection for objective 3. (PDF 173 kb) [file 12905_2017_486_MOESM4_ESM.pdf]

AUDIOLOGICAL PROCEDURES TO BE UTILIZED DURING DATA COLLECTION FOR OBJECTIVE 3

**Audiological Test, Procedure and Duration**	**Motivation**	**Tool**
**Interview**<br>Procedure: The interview will be conducted by the researcher<br><br>Duration: ± 5 minutes	The purpose of obtaining case history information will be to aid in establishing an overview of the participant's auditory status, medical conditions, medication, noise exposure and communicative abilities related to audition [1]. Information on the risk factors for ototoxic hearing loss will, thus, also be documented during the case history interview. This questionnaire will also include questions on tinnitus, as a common complaint of cancer patients on chemotherapy is the onset of tinnitus.	Case history form, focusing on hearing history, medical history, family history, and history of noise exposure, was developed. Questions on tinnitus were adapted from the Tinnitus Ototoxicity Monitoring Interview (TOMI) schedule [2].
**Otoscopic Examinations**<br>Procedure: The researcher will look into the patient's ear canal using an otoscope.<br>Duration: ± 2 minutes	An otoscopic examination of both ears will be conducted to determine the status of the tympanic membrane and the external ear [3].	An Agine otoscope will be used to conduct otoscopic examinations.
**Immittance Audiometry-Tympanometry and ipsilateral and contra-lateral acoustic reflex threshold testing**<br>Procedure: A soft probe will	Tympanometry will be conducted to determine if there are any abnormalities in the middle ear as well as to aid in detection and differentiation of possible pathologies [1].<br>The acoustic reflex threshold is the lowest intensity of a sound stimulus that elicits a measurable change in the acoustic admittance [4]. "These measures provide information related to the function of the middle ear and the sensory, neural, and motor pathways associated with the reflex arc"[5] (p. 169). Both ipsilateral and	A clinical impedance audiometer, the GSI Tympstar V2 Impedance meter will be used for immittance audiometry.

be placed in the entrance of the participant's ear canal and will introduce slight pressure as well as soft beeping sounds. This test does not cause any discomfort and the participant will not be required to respond.<br>Duration: ± 5 minutes	contra-lateral reflex testing will be conducted as this will help identify individuals with possible cochlear, retro-cochlear and brainstem pathology.	
**Pure Tone Audiometry**<br>**(air and bone conduction)**<br>Procedure: Headphones will be placed on the participants' ears and they will hear a beeping sound, where they will be required to press a button to indicate that they heard the sound.<br><br>Duration: ± 20 minutes	Since pure tone audiometry is the foundation of every audiological evaluation, its' results will form the basis from which an initial diagnosis of hearing loss can be made [6]. It will be conducted to also quantify the degree of hearing loss and determine the type of hearing loss [7].<br><br>Pure tone air conduction thresholds will be obtained bilaterally via air conduction at the following frequencies: 125, 250, 500, 1000, 2000, 4000, 6000, 8000, 9000, 10000, 11200, 12500, 14000, 16000, 18000 and 20000 Hz, while bone conduction thresholds will be obtained bilaterally at 250, 500, 1000, 2000 and 4000Hz.<br><br>Bone conduction audiometry will be conducted at baseline, post treatment audiometry 6 months after completion of chemotherapy and if there is a significant change in the air conduction thresholds during the other audiological evaluations.	A twin channel clinical diagnostic audiometer, the Madsen Astera will be used for pure tone audiometry.

**Speech Audiometry**<br>Procedure: Headphones will be placed on the participants' ears and they will hear words which they will be required to repeat.<br><br>Duration: ± 10 minutes	Speech recognition threshold (SRT) testing will be conducted to determine the lowest hearing level at which spondaic words are identified 50% of the time [3]. It is also a tool used for the confirmation of pure tone thresholds and in doing so, alerts the audiologist to invalid pure tone results [8].<br><br>Speech recognition score testing is conducted to measure how well the listener can understand speech as a function of the ability to differentiate sounds under optimum circumstances. The score is intended to be a measure of the clarity with which the patient hears speech [8]. Speech audiometry will be conducted for the baseline audiological evaluation and the 6 month follow-up evaluation.	SRT- The CID Spondee word list will be used. For isiZulu speakers, the digits test, will be used, as it is low linguistically loaded [9].<br>SRS- The CID W-22 Auditory test word list will be used. For isiZulu speakers, an isiZulu wordlist collated in the Discipline of Audiology, will be used.<br>The audiometric sound proof booth and the twin channel audiometer, as described for pure tone audiometry will be used.
**Distortion Product Oto-Acoustic Emission Testing**<br>Procedure: A soft probe will be placed in the entrance of the participant's ear canal and will introduce soft beeping sounds. This test does not cause any discomfort and the participant will not be required to respond.<br>Duration: ± 5 minutes	"OAEs are believed to be the by-products of the preneural mechanisms of the cochlear amplifier, and in particular to be linked to the normal functioning of the outer hair cells"[10] (p. 441). Therefore, OAEs are sensitive to hearing losses, resulting from outer hair cell damage. OAE results will help with the differentiation of cochlear vs. retrocochlear disorders and also identify individuals with subtle abnormalities of CNS function [11].	The Mico Oto–acoustic emissions will be used for the elicitation of the OAE.

References

[1] Gelfand SA. Essentials in audiology. New York: Thieme Medical Publishers, Inc; 1997.

[2] Fausti SA, Helt WJ, Gordon JS. Audiologic monitoring for ototoxicity and patients management. In Campbell KC, editor. Pharmacology and ototoxicity for audiologists. United States: Thomson Delmar Learning. 2007. p. 230-251.

[3] Martin FN, Clark J. Introduction to audiology. 10th ed. Boston: Pearson; 2009.

[4] Gelfand SA. The acoustic reflex: In Katz J, editor. Handbook of clinical audiology. 5th ed. Baltimore: Lippincott Williams and Wilkins. 2002. p. 205-33.

[5] Wiley TL, Stoppenbach DT. Basic principles of acoustic immittance measures. In Katz J, editor. Handbook of clinical audiology. 5th ed. Baltimore: Lippincott Williams and Wilkins. 2002. p. 159-74.

[6] Roeser RJ, Buckney KA, Stickney GS. Pure tone tests. In Roeser RJ, Valente M, Hosford-Dunn H, editors. Audiology diagnosis. New York: Thieme Medical Publishers. 2000. p. 227-52.

[7] Harrell RW. Pure tone evaluation. In Katz J, editor. Handbook of clinical audiology. 5th ed. Baltimore: Lippincott Williams and Wilkins. 2002. p. 71-87.

[8] Hodgson WR. Basic audiological evaluation. Baltimore: Williams and Wilkins. 1980.

[9] Ramkissoon I. Digit speech recognition thresholds (SRT) for non-native speakers of English. Am J Audiol. 2002; 11: 23-28.

[10] Prieve BA, Fitzgerald TS. Otoacoustic emissions. In Katz J, editor. Handbook of clinical audiology. 5th ed. Baltimore: Lippincott Williams and Wilkins; 2002. p. 440-68.

[11] Robinette MS, Glattke TJ. Otoacoustic emissions. In Roeser RJ, Valente M, Hosford-Dunn H, editors. Audiology diagnosis. New York: Thieme Medical Publishers; 2000. p. 503-26.
