# Supplementary material for: Research protocol: Cisplatin-associated ototoxicity amongst patients receiving cancer chemotherapy and the feasibility of an audiological monitoring program
Source: BMC Womens Health. 2017 Dec 11;17:129. doi: 10.1186/s12905-017-0486-8 (PMC5725900; doi:10.1186/s12905-017-0486-8)
Supplement: Supplementary file 5 — Interview questionnaire for patients. (PDF 103 kb) [file 12905_2017_486_MOESM5_ESM.pdf]

Cisplatin-associated ototoxicity amongst patients receiving cancer chemotherapy and the feasibility of an audiological monitoring program

INTERVIEW QUESTIONNAIRE FOR PATIENTS

DATE: [ ]

1. PERSONAL INFORMATION

CODE [ ]

BIRTHDATE: [ ] AGE: [ ]

RACE [ ] GENDER: [ ]

AUDIOLOGICAL EVALUATION: BASELINE: [ ] CYCLE 4: [ ]

FOLLOW UP: 1 [ ] 3 [ ] 6 [ ]

2. HEARING HISTORY

2.1. Do you experience hearing difficulties? Yes No

2.2. When did you first notice the problem?

2.3. In which ear do you experience these difficulties? Left Right Both

2.4. Do you experience any pain in your ears? Yes No

2.5. Do you hear noises in your ears or head? Yes No

2.5.1. How long have you been hearing these noises?

	< 1 week	1 week
2 weeks	1 month	2 months
3 months	< 6 months	6 months
< 1 year	>1 year	

2.5.2. Where is the noise present?

Head	Left Ear	Right ear	Both ears

2.5.3. Describe the sound.

High pitched	Low pitched	Ringing	Pulsating	Roaring

2.5.4. When do you hear this sound?

Morning	Midday	Afternoon	Night
All the time			

2.5.5. Is it present?

Continuously	Intermittently

2.5.6. Describe the loudness of these noises

Not loud at all	Slightly loud	Moderately loud	Very loud

**2.5.7. Has these noises changed since you started the treatment

Yes	No

**2.5.8. Has the loudness of these noises changed?

Yes, louder now	Yes, quieter now	No

2.6. Do you ever have a feeling of fullness or stuffiness in your ears?

Yes	No

---

3. OTOLOGIC HISTORY

3.1. Have you ever had any repeated ear infections?

Yes	No

3.1.1. Which ear?

Right	Left	Both

3.2. Have you had any surgery on your ears?

Yes	No

3.2.1. If yes, please specify what surgery as well as the date. DATE

SURGERY

3.3.Have you ever had any injury to the head or neck region?

Yes	No

3.4.If so, provide details

---

4. FAMILY HISTORY

4.1. Does anyone in your family have a hearing loss? Yes No

4.2. If so, who?

4.3.What was the cause of the hearing loss?

5. GENERAL MEDICAL HISTORY (To be completed in conjunction with review of patients medical records)

5.1. Do you suffer from any other medical conditions? Yes No

5.2. Name the conditions.

5.3. Have you ever been diagnosed with Tuberculosis (TB) and/or malaria?

Yes No

5.4. When were you diagnosed with cancer?

5.5. Did you receive any other treatment for the cancer? Yes No

5.5.1. If so, what treatment did you receive? Surgery | Radiation therapy | Both

MEDICAL REVIEW

5.6. Type of cancer: ____

5.7. Stage of cancer: ____

5.8. List all the medication and dosage.

Medication	Dosage

5.9. Renal function: ____

5.10. Current weight: ____

6. NOISE EXPOSURE HISTORY

6.1. Have you ever been exposed/or are exposed to loud noise for long periods of time?

Yes	No

6.2. If yes, please specify the type of noise?

** - denotes questions to be asked if the patient had experienced tinnitus prior to commencement of chemotherapy.
