# Supplementary material for: Research protocol: Cisplatin-associated ototoxicity amongst patients receiving cancer chemotherapy and the feasibility of an audiological monitoring program
Source: BMC Womens Health. 2017 Dec 11;17:129. doi: 10.1186/s12905-017-0486-8 (PMC5725900; doi:10.1186/s12905-017-0486-8)
Supplement: Supplementary file 6 — Audiogram. (PDF 702 kb) [file 12905_2017_486_MOESM6_ESM.pdf]

DISCIPLINE OF AUDIOLOGY

AUDIOLOGICAL EVALUATION RECORDING FORM

PARTICIPANT CODE: ____________________

TEST DATE: ___________________________ TEST NO.: __________________

TEST RELIABILITY: ______________________

PURE TONE AVERAGE: RIGHT _________ LEFT_____________

UNIVERSITY OF KWAZULU-NATAL
INYUVESI YAKWAZULU-NATALI

PURE TONE AUDIOMETRY

RIGHT

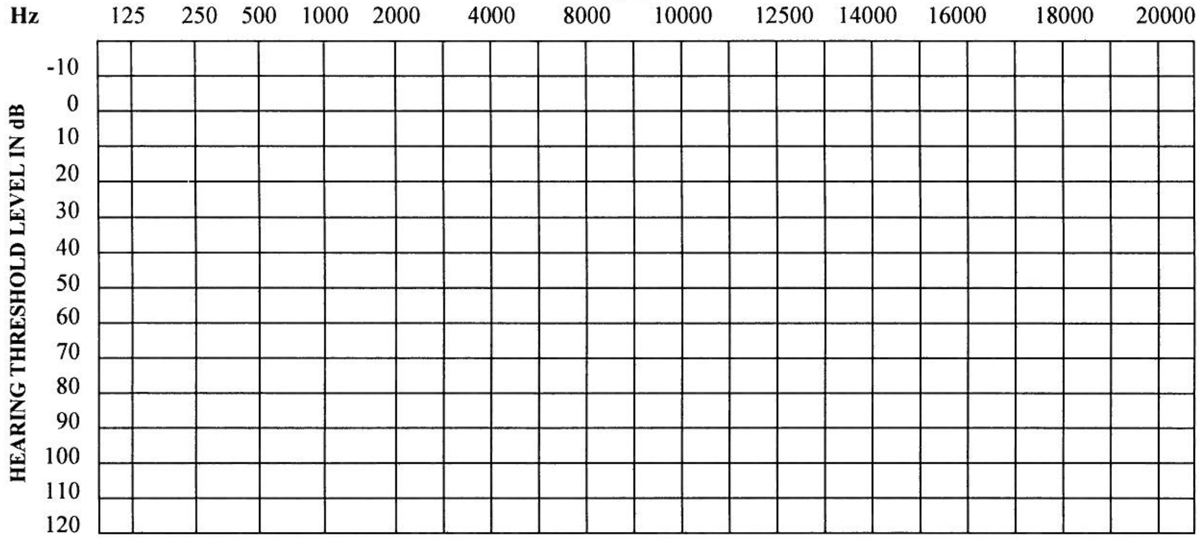

Masking Levels on Non-Test Ear. **Type of masking:**______________

Hz	250	500	1k	2k	4k	8k
AC						
BC						

LEFT

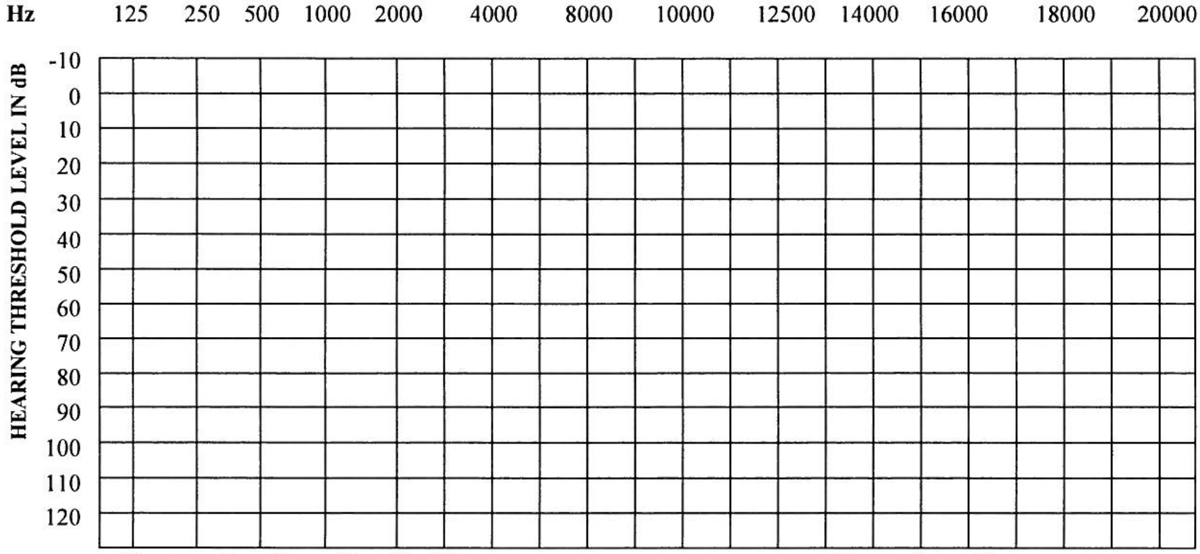

Masking Levels on Non-Test Ear. **Type of masking:**____________

Hz	250	500	1k	2k	4k	8k
AC						
BC						

STIMULUS	Left Blue	Right Red	Left Blue	Right Red
Air Conduction<br>AC Masked	×<br>□	O<br>△	×<br>□	O<br>△
Bone Conduction	>	<	>	<
B/C Masked	]	[	]	[
AR – Contra<br>AR - Ipsi	-<br>-	-<br>-	↴<br>↴	⤵<br>⤵

SPEECH AUDIOMETRY

SPEECH RECOGNITION THRESHOLD

EAR	SRT	MASKING	
		LEVEL	EAR
RIGHT			
LEFT			

SPEECH DISCRIMINATION SCORE

EAR	dBHL	%	MASKING	
			LEVEL	EAR
RIGHT				
LEFT				

P.I. FUNCTION

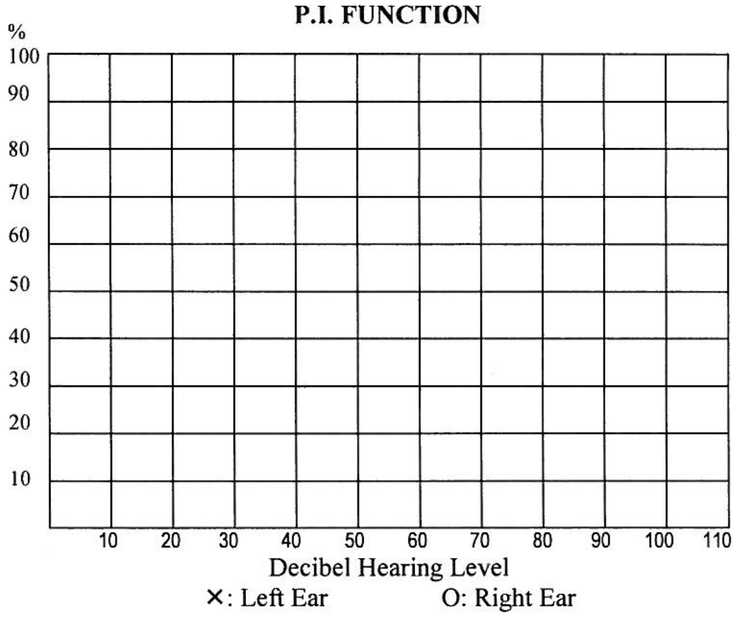

IMMITTANCE AUDIOMETRY

Tympanometry

Right		Left
	**Ear canal volume**	
	**Middle ear pressure**	
	**Static compliance**	
	**Type**	

Acoustic Reflex Threshold Testing

Right		Frequency	Left	
Ipsilateral Reflex	Contralateral Reflex		Ipsilateral Reflex	Contralateral Reflex
	-	**250Hz**		-
		**500Hz**		
		**1000Hz**		
		**2000Hz**		
		**4000Hz**		

DISTORTION PRODUCT OTO-ACOUSTIC EMISSIONS

Right	GM	Left
	**7253**	
	**5130**	
	**3616**	
	**2589**	
	**1818**	
	**1305**	
	**933**	
	**676**	

OTOSCOPIC EXAMINATION

RIGHT EAR	OBSERVATION	LEFT EAR
	**Outer Ear**	
	**Ear canal**	
	**Tympanic Membrane**	
	**Surrounding Structures**	
