# Supplementary material for: Research protocol: Cisplatin-associated ototoxicity amongst patients receiving cancer chemotherapy and the feasibility of an audiological monitoring program
Source: BMC Womens Health. 2017 Dec 11;17:129. doi: 10.1186/s12905-017-0486-8 (PMC5725900; doi:10.1186/s12905-017-0486-8)
Supplement: Supplementary file 7 — Measurements and equipment for audiological evaluations. (PDF 177 kb) [file 12905_2017_486_MOESM7_ESM.pdf]

MEASUREMENTS AND EQUIPMENT FOR AUDIOLOGICAL EVALUATIONS

Audiometric results will be analysed according to the following guidelines indicated in literature.

1. Otoscopic Examination

The different structures of the ear i.e. the pinna, ear canal, tympanic membrane and surrounding structures will be commented on.

a) The pinna will be described as normal, abnormal and presenting with sores.

b) The ear canal will be described as normal, reddened, or presenting with sores, discharge, foreign bodies, excessive wax, impacted wax, or blood in the ear canal.

c) The tympanic membrane will be described as intact, retracted, perforated, or reddened.

d) The surrounding structures will be described as normal, presenting with pre auricular swelling and/or swollen mastoid.

2. Immittance Audiometry

2.1. Tympanometry

Tympanometric tracings will be compared to Jerger's classification[1]:

a) A Type A tympanogram is obtained when static compliance (0.2 – 2.0 ml), middle ear pressure (-100 to 100daPa) and ear canal volume (0.2 to 1.8 ml) are within normal limits and this is seen in normal hearing individuals and those with sensorineural hearing loss. Tympanograms with normal middle ear pressure and ear canal volume but low static compliance is termed type As and is seen in individuals with otosclerosis. On the other hand, tympanograms with normal middle ear pressure and ear canal volume but high static compliance is termed type Ad and is seen in normal hearing patients with highly flaccid tympanic membranes.

b) A Type B tympanogram is obtained when ear canal volume is normal but there is no peak pressure. This is seen in individuals with middle ear dysfunction. A Type B tympanogram with ear canal volume lower than 0.2 ml and no peak pressure is seen in wax occlusion, while type B tympanogram with ear canal volume higher than 1.8 ml and no peak pressure is seen in patients with tympanic membrane perforation or patent ventilation tubes.

c) A Type C tympanogram is obtained when ear canal volume and static compliance is normal but peak pressure is less than -100daPa. This is seen in patients with Eustachian tube dysfunction and retracted tympanic membranes.

2.2. Acoustic reflex threshold testing

Measurements will be based on normative data for the contralateral (70dBSL – 95dBSL as suggested by Metz, 1952 [2] and ipsilateral (3- 6 dB SL better than contralateral as suggested by Moller, 1962 & Fria et al., 1975 [3]) acoustic reflexes.

3. Pure Tone Audiometry

For air conduction pure tone audiometry, measurements will based on the thresholds at various frequencies i.e. 125Hz, 250Hz, 500Hz, 1000Hz, 2000Hz, 4000Hz, 8000Hz, 9000Hz, 10000Hz, 11200Hz, 12000Hz, 14000Hz, 16000Hz, 18000Hz, and 20000Hz.

Hearing loss will be classified according to ANSI standards [4], as follows:

Classification of Hearing Loss

**Decibel Level**	**Degree of hearing loss**
-10 to 15	Normal
16 to 25	slight
26 to 40	mild
41 to55	Moderate
56 to 70	Moderately severe
71 to 90	Severe
>90	Profound

The same normative data will also apply to frequencies above 8000Hz, as there is adequate reliability within clinically acceptable limits and accurate thresholds can be obtained up to 20 000Hz [5]. Furthermore, including test frequencies from 9 to 20 kHz provides maximum hearing sensitivity information with which to monitor hearing change [6]; therefore, frequencies 18000 and 20000Hz were included. While the utility of testing frequencies above 16000Hz is questionable, it has been included as the study is longitudinal in nature with the baseline assessment of each patient serving as his own reference.

For bone conduction pure tone audiometry, measurements will be based on thresholds from 500Hz to 4000Hz. A comparison of the air and bone conduction threshold helps with determining the

type of hearing loss as follows: a) air and bone conduction thresholds within normal limits indicates normal peripheral hearing, b) air and bone conduction depressed in the absence of an air-bone-gap indicates sensorineural hearing loss, c) air and bone conduction depressed in the presence of an air-bone-gap indicates mixed hearing loss [7].

The following would represent a significant hearing loss [6]:

(a) ≥ 20dB decrease at any one test frequency,

(b) ≥10dB decrease at any two adjacent frequencies, or

(c) loss of response at three consecutive frequencies where responses were previously obtained.

4. Speech audiometry

The researcher will obtain both speech recognition threshold (SRT) and speech recognition scores (SRS).

4.1. Speech recognition threshold

A comparison of the SRT and Pure tone average would be conducted. If a value of 0-10dB is obtained, then the result for the pure tone assessment is valid [8].

4.2. Speech recognition score

Speech recognition scores will be not be evaluated using a guide. Rather, the patient's SRS from subsequent evaluations will be compared to the baseline and the preceding audiological evaluation. A guideline cannot be used as the isiZulu wordlist has not been normed or validated, and therefore the guideline for the English wordlists cannot be used for isiZulu speaking participants. The P.I. function will not be evaluated, as the isiZulu wordlists do not contain phonetically balanced words.

5. Oto-acoustic emissions,

Measurements will be based on a difference between the DPOAE and the individual's noise floor in the frequency range of 500Hz and 8000Hz. An emission will be considered present if the difference is greater than 6dB and the absolute amplitude greater than -10dB SPL [9].

AUDIOLOGICAL PROCEDURE AND EQUIPMENT

All equipment was calibrated by a qualified technician, whilst the researcher conducted daily biological calibrations prior to data collection.

Audiological Test, Procedure and Duration	Tool
**Otoscopic Examinations**<br>Procedure: The researcher will look into the patient's ear canal using an otoscope.<br>Duration: Approximately 2 minutes	An Agine otoscope will be used to conduct otoscopic examinations.
**Immittance Audiometry- Tympanometry and ipsilateral and contra-lateral acoustic reflex threshold testing**<br>Procedure: A soft probe will be placed in the entrance of the participant's ear canal and will introduce slight pressure as well as soft beeping sounds. This test does not cause any discomfort and the participant will not be required to respond.<br><br>Duration: Approximately 5 minutes	A clinical impedance audiometer, the GSI Tympstar V2 Impedance meter, which is calibrated annually will be used for immittance audiometry.
**Pure Tone Audiometry**<br>**(air and bone conduction)**<br>Procedure: Headphones will be placed on the participants' ears and they will hear a beeping sound, where they will be required to press a button to indicate that they heard the sound.<br><br>Duration: Approximately 20 minutes	An audiometric sound proof booth of double wall construction, meeting the noise level requirements as set by ANSI (2004), will be used as the test environment for pure tone testing. A twin channel clinical diagnostic audiometer, the Madsen Astera, which is calibrated annually, to meet the standards set by SABS, will be used for pure tone audiometry.
**Speech Audiometry**<br>Procedure: Headphones will be placed on the participants' ears and they will hear words which they will be required to repeat.	SRT- The CID Spondee word list will be used. For isiZulu speakers, the digits test, will be used, as it is low linguistically loaded.<br>SRS- The CID W-22 Auditory test word list will be used. For isiZulu speakers, an isiZulu wordlist,

Duration: approximately 10 minutes	collated in the Discipline of Audiology, will be used.<br>The twin audiometric sound proof booth and the twin channel audiometer, as described for pure tone audiometry will be used.
**Distortion Product Oto-Acoustic Emission Testing**<br>Procedure: A soft probe will be placed in the entrance of the participant's ear canal and will introduce soft beeping sounds. This test does not cause any discomfort and the participant will not be required to respond.<br><br>Duration: Approximately 5 minutes	The Madsen Capella Oto–acoustic emissions, calibrated annually, will be used for the elicitation of the OAE.

References

[1] Jerger J. Clinical experience with impedance audiometry. Arch Otolaryngol. 1970; 92: 311-24.

[2] Feldman AS. Acoustic impedance-admittance battery. In Katz J, editor. Handbook of clinical audiology. 2nd ed. Baltimore: Williams and Wilkins Company; 1978. p. 356-74.

[3] Northern JL, Grimes AM. Introduction to acoustic impedance. In Katz J, editor. Handbook of clinical audiology. 2nd ed. Baltimore: Williams and Wilkins Company; 1978. p. 344–55.

[4] American National Standards Institute (ANSI). American National Standards specification for audiometers. ANSI S3.6. New York; 2004.

[5] Bieter RC, Talley JN. High Frequency Audiometry. Audiology. 1976; 15: 207 –14.

[6] American Speech-Language-Hearing Association. Audiologic management of patients receiving cochleotoxic drug therapy [guidelines]. www.asha.or/policy (1994). Accessed 30 January 2010

[7] Martin FN, Clark J. Introduction to audiology. 10th ed. Boston: Pearson; 2009.

[8] Kramer SJ. Audiology: Science to practice, San Diego: Plural Publishing, Inc; 2008.

[9] Hall JW. Handbook of Otoacoustic Emissions. San Diego: Singular Publishing; 2000.
