# Supplementary material for: Research protocol: Cisplatin-associated ototoxicity amongst patients receiving cancer chemotherapy and the feasibility of an audiological monitoring program
Source: BMC Womens Health. 2017 Dec 11;17:129. doi: 10.1186/s12905-017-0486-8 (PMC5725900; doi:10.1186/s12905-017-0486-8)
Supplement: Supplementary file 8 — Interview Questionnaire for oncology clinic personnel and pharmacists post ototoxicity program. (PDF 158 kb) [file 12905_2017_486_MOESM8_ESM.pdf]

Cisplatin-associated ototoxicity amongst patients receiving cancer chemotherapy and the feasibility of an audiological monitoring program

INTERVIEW QUESTIONNAIRE FOR ONCOLOGY CLINIC PERSONNEL AND PHARMACISTS POST OTOTOXICITY PROGRAM

Dear Participant

We are delighted that you have agreed to participate and would like to thank you sincerely, for the information from this study can be used to help us understand the complexities associated with chemotherapy. The information that you provide will be treated with the strictest of confidence and please do not hesitate to ask us any questions that you may have during the course of the study. Contact details are reflected on the information and consent document.

INSTRUCTIONS

1. Please mark the appropriate answer to each question with an X, and give further detail if necessary.

2. Please answer all questions.

1. How many patients with cervical cancer did you treat/dispense cisplatin medication to, during the study period?

< 20	20-49	50-69	70-100

2. Did you notice any changes in the patient's hearing?

Yes	No	Sometimes

3. Do you provide patients with information regarding the ototoxic effects of medication?

Yes	No	Sometimes

4. Do you provide patients with any information regarding their hearing?

Yes	No	Sometimes

5. Do you enquire about patient's history of hearing difficulties?

Yes	No	Sometimes

6. How many referrals have you made to the audiologist over the last year?

<20	20-49	50-69	70-100

7. How many hours in a day are spent on the ototoxicity monitoring program?

< 2 hours	2- <4 hours	4-<6 hours	6-8 hours

8. Do you feel that the ototoxicity monitoring program is beneficial?

Yes	No

8.1. Why?

____

____

8.2. Was the team approach to managing the patient with cancer successful?

Yes	No	Sometimes

8.2.1. Discuss

____

____

____

8.2.2. Describe the collaboration with the audiologist and other team members.

____

____

____

----------------------------THANK YOU FOR YOUR TIME AND COOPERATION------------------
