# Supplementary material for: Research protocol: Cisplatin-associated ototoxicity amongst patients receiving cancer chemotherapy and the feasibility of an audiological monitoring program
Source: BMC Womens Health. 2017 Dec 11;17:129. doi: 10.1186/s12905-017-0486-8 (PMC5725900; doi:10.1186/s12905-017-0486-8)
Supplement: Supplementary file 9 — Questionnaire for participants with cancer post monitoring program. (PDF 154 kb) [file 12905_2017_486_MOESM9_ESM.pdf]

Cisplatin-associated ototoxicity amongst patients receiving cancer chemotherapy and the feasibility of an audiological monitoring program

QUESTIONNAIRE FOR PARTICIPANTS WITH CANCER POST MONITORING PROGRAM

Dear Participant

We are delighted that you have agreed to participate and would like to thank you sincerely, for the information from this study can be used to help us understand the complexities associated with chemotherapy. The information that you provide will be treated with the strictest of confidence and please do not hesitate to ask us any questions that you may have during the course of the study. Contact details are reflected on the information and consent document.

INSTRUCTIONS

1. Please mark the appropriate answer to each question with an X, and give further detail if necessary.

2. Please answer all questions.

1. Did you receive information about the effects of the chemotherapy medication before commencing with treatment?

Yes	No

2. If so, who provided you with information?

Nurse	Oncologist	Pharmacist	Audiologist

3. Who referred you for the audiological monitoring?

Nurse	Oncologist	Pharmacist	Audiologist

4. Were the audiological evaluations conducted on the same day as your chemotherapy?

Yes	No	Sometimes

5. Do you feel that the duration of the audiological testing was too long?

Yes	No

6. Were the results of the audiological evaluations clearly explained to you?

Yes	No	Sometimes

7. Which of the following recommendations were made?

Hearing aid evaluation	Counselling	Tinnitus management

8. Did you follow-up on any of the above recommendations made?

Yes	No	Sometimes

9. Do you feel that the monitoring of your hearing during chemotherapy was beneficial?

Yes	No	Don’t know
