# Supplementary material for: Research protocol: Cisplatin-associated ototoxicity amongst patients receiving cancer chemotherapy and the feasibility of an audiological monitoring program
Source: BMC Womens Health. 2017 Dec 11;17:129. doi: 10.1186/s12905-017-0486-8 (PMC5725900; doi:10.1186/s12905-017-0486-8)
Supplement: Supplementary file 10 — Ethical Approval from University of Kwazulu-Natal. (PDF 24 kb) [file 12905_2017_486_MOESM10_ESM.pdf]

UNIVERSITY OF
KWAZULU-NATAL
INYUVESI
YAKWAZULU-NATALI
RESEARCH OFFICE
BIOMEDICAL RESEARCH ETHICS ADMINISTRATION
Westville Campus
Govan Mbeki Building
Private Bag X 54001
Durban
4000
KwaZulu-Natal, SOUTH AFRICA
Tel: 27 31 2604769 - Fax: 27 31 2604609
Email: BREC@ukzn.ac.za
Website: http://research.ukzn.ac.za/Research-Ethics/Biomedical-Research-Ethics.aspx

07 July 2014

Ms. J Paken
Department of Audiology
School of Health Sciences
University of KwaZulu-Natal

**PROTOCOL: Cisplatin-associated ototoxicity amongst patients with lung cancer and the feasibility of an audiological monitoring program at Grey's Hospital. REF: BE064/13.**

I wish to advise that your application for Amendments dated 19 June 2014 for the above study has been noted and approved by a sub-committee of the Biomedical Research Ethics Committee. The letter from School Academic leader dated 20 June 2014 supporting this request has been noted by BREC.

BREC has approved the change of title to the following:

**"Cisplatin-associated ototoxicity amongst patients receiving cancer chemotherapy and the feasibility of an audiological monitoring program at Grey's Hospital"**

The approval will be noted by a full Committee at a meeting to be held on 12 August 2014.

Yours sincerely

Ms A Marimuthu
Senior Admin Officer: Biomedical Research Ethics
