# Supplementary material for: Research protocol: Cisplatin-associated ototoxicity amongst patients receiving cancer chemotherapy and the feasibility of an audiological monitoring program
Source: BMC Womens Health. 2017 Dec 11;17:129. doi: 10.1186/s12905-017-0486-8 (PMC5725900; doi:10.1186/s12905-017-0486-8)
Supplement: Supplementary file 11 — Funding approval from South African Medical Research Council. (PDF 292 kb) [file 12905_2017_486_MOESM11_ESM.pdf]

SOUTH AFRICAN MEDICAL RESEARCH COUNCIL

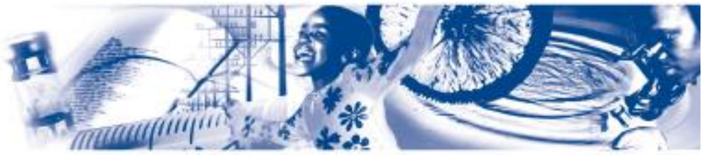

Research Capacity Development

Sub Directorate

PO Box 19070, Tygerberg 7505, South Africa
Francie van Zijl Drive, Parow Valley, Cape Town
Tel +27 21 938 0911/938 0437, Fax +27 21 938 0377

23 February 2013

Dear Jessica Paken

It is my pleasure to inform you that your application for PhD funding: National Health Scholars Programme (NHSP) 2013 has been successful. We will be in touch with you for further detail regarding contract signing and other official processes. As advertised, the funding - soon be confirmed in a contract, will be commensurate with your current level of cost to company for your qualification(s).

Further and conclusive detail will appear in the forthcoming contract, currently being processed at MRC Legal. You will be able to accept or reject the offer then for us to proceed.

Congratulations again.

Regards.

T. Maitin

Dr T Maitin
Division Manager: Research Capacity Development
Medical Research Council of South Africa
TEL: 021 938 0891; FAX: 021 938 0377
E-mail: thabi.maitin@mrc.ac.za
