# Supplementary material for: Research protocol: Cisplatin-associated ototoxicity amongst patients receiving cancer chemotherapy and the feasibility of an audiological monitoring program
Source: BMC Womens Health. 2017 Dec 11;17:129. doi: 10.1186/s12905-017-0486-8 (PMC5725900; doi:10.1186/s12905-017-0486-8)
Supplement: Supplementary file 12 — Information document for clinic personnel. (PDF 197 kb) [file 12905_2017_486_MOESM12_ESM.pdf]

**<u>INFORMATION DOCUMENT FOR CLINIC PERSONNEL</u>**

**DISCIPLINE OF AUDIOLOGY**
**SCHOOL OF HEALTH SCIENCES**
**Tel: 031 260 7438/8986**
**Fax: 031 260 7622**
**E-mail: sitholep2@ukzn.ac.za**
**E-mail: naidoor1@ukzn.ac.za**

UNIVERSITY OF KWAZULU-NATAL
INYUVESI YAKWAZULU-NATALI

---

**<u>Cisplatin-induced ototoxicity amongst patients with ovarian cancer and the feasibility of an audiological monitoring program at the Inkosi Albert Luthuli Central Hospital</u>**

To whom it may concern

I, Jessica Paken, under the supervision of Prof V. Sewram and Mr. C. D. Govender, am doing research on cancer chemotherapy and resultant hearing loss. In the study, I want to determine if patients on cancer chemotherapy develop a hearing loss.

Since you meet the criteria to be a participant in this study i.e., employed within the oncology unit for at least two months, I hereby invite you to participate in this study.

If you choose to participate in this study, you will be required to complete a questionnaire, which should take approximately 10 minutes to complete. Your participation will help increase the understanding of audiological related outcomes in patients on cancer chemotherapy.

This is a risk free procedure; therefore no harm would come to you. Your participation is voluntary and you can withdraw at any point. All information from the research will be kept strictly confidential. All results will be presented in the study with codes and numbers, with no reference to names and the data will be presented to research supervisors in a similar manner.

Should you have any further enquiries or concerns, you may contact me (Tel. No.: 031-2607548 or 084 2424 005 or e-mail: pakenj@ukzn.ac.za) or the
BIOMEDICAL RESEARCH ETHICS ADMINISTRATION
University of KwaZulu-Natal
Research Office, Westville Campus
Govan Mbeki Building
Private Bag X 54001, Durban, 4000
KwaZulu-Natal, SOUTH AFRICA
Tel: 27 31 2604769 - Fax: 27 31 2604609
Email: BREC@ukzn.ac.za.

Yours sincerely

| ______________________ | ______________________ | ______________________ |
|---|---|---|
| Jessica Paken<br>(Principal Investigator) | Prof V. Sewram<br>(Research Supervisor) | Mr C. D. Govender<br>(Research Co- supervisor) |
