# Supplementary material for: Research protocol: Cisplatin-associated ototoxicity amongst patients receiving cancer chemotherapy and the feasibility of an audiological monitoring program
Source: BMC Womens Health. 2017 Dec 11;17:129. doi: 10.1186/s12905-017-0486-8 (PMC5725900; doi:10.1186/s12905-017-0486-8)
Supplement: Supplementary file 13 — Information document for patients. (PDF 308 kb) [file 12905_2017_486_MOESM13_ESM.pdf]

INFORMATION DOCUMENT FOR PATIENTS

DISCIPLINE OF AUDIOLOGY
SCHOOL OF HEALTH SCIENCES
Tel: 031 260 7438/8986
Fax: 031 260 7622
E-mail: sitholep2@ukzn.ac.za
E-mail: naidoor1@ukzn.ac.za

UNIVERSITY OF KWAZULU-NATAL
INYUVESI YAKWAZULU-NATALI

INFORMATION DOCUMENT

Cisplatin-induced ototoxicity amongst patients with ovarian cancer and the feasibility of an audiological monitoring program at the Inkosi Albert Luthuli Central Hospital

To whom it may concern

I, Jessica Paken, under the supervision of Prof V. Sewram and Mr. C. D. Govender, am doing research on cancer chemotherapy and resultant hearing loss. In the study, I want to determine if patients on cancer chemotherapy develop a hearing loss. This does not form part of your treatment but will help in determining your hearing status.

Since you meet the criteria to be a participant in this study i.e. over the age of 18, diagnosed with ovarian cancer and commencing with chemotherapy, I would like to invite you to participate in this study.

If you choose to participate in this study, I will access your file to determine the name and dosage of your medication. In addition, you will be interviewed about your hearing history. You will also be required to undergo a **PAIN-FREE** hearing evaluation. This would entail;

1. I would look into your ear canal with an otoscope.
2. A soft probe will be placed in the entrance of your ear canal and you will feel a bit of pressure and also hear a sound. You will not be required to respond.
3. Headphones will be placed on your ears and you will hear a beeping sound, where you will be required to press a button to indicate that you heard the sound.
4. Headphones will be placed on your ears and you will hear words spoken to you, where you will be required to repeat the words.

The entire procedure will last no longer than 45 minutes, but will be conducted before you receive each cycle of chemotherapy and at one, three and six months after chemotherapy. This is a risk free procedure; therefore no harm would come to you. Your participation is voluntary and you can withdraw at any point.

Benefits of participation include a free hearing evaluation. Should you fail the hearing evaluation; appropriate referrals will be made. Complete participation will contribute to acquiring important clinical data for the hearing test interpretation.

You will not be required to pay for any services. You will be reimbursed R100.00 for “out of pocket” expenses.

For the purpose of transcribing your results, your results will be printed. However, all information from the research will be kept strictly confidential. All results will be presented in the study with codes and numbers, with no reference to names and the data will be presented to research supervisors in a similar manner.

An individual who speaks your language will be available to address your concerns on the day of the test.

Should you have any further enquiries or concerns, you may contact me (Tel. No.: 031-2607548 or 084 2424 005 or e-mail: pakenj@ukzn.ac.za) or the

BIOMEDICAL RESEARCH ETHICS ADMINISTRATION
University of KwaZulu-Natal
Research Office, Westville Campus
Govan Mbeki Building
Private Bag X 54001, Durban, 4000
KwaZulu-Natal, SOUTH AFRICA
Tel: 27 31 2604769 - Fax: 27 31 2604609
Email: BREC@ukzn.ac.za.

Yours sincerely

____
Jessica Paken
(Principal Investigator)

____
Prof V. Sewram
(Research Supervisor)

____
Mr C. D. Govender
(Research Co- supervisor)
