# Supplementary material for: Research protocol: Cisplatin-associated ototoxicity amongst patients receiving cancer chemotherapy and the feasibility of an audiological monitoring program
Source: BMC Womens Health. 2017 Dec 11;17:129. doi: 10.1186/s12905-017-0486-8 (PMC5725900; doi:10.1186/s12905-017-0486-8)
Supplement: Supplementary file 14 — Consent document for clinic personnel. (PDF 196 kb) [file 12905_2017_486_MOESM14_ESM.pdf]

CONSENT DOCUMENT FOR CLINIC PERSONNEL

DISCIPLINE OF AUDIOLOGY
SCHOOL OF HEALTH SCIENCES
Tel: 031 260 7438/8986
Fax: 031 260 7622
E-mail: sitholep2@ukzn.ac.za
E-mail: naidoor1@ukzn.ac.za

CONSENT DOCUMENT

Cisplatin-induced ototoxicity amongst patients with ovarian cancer and the feasibility of an audiological monitoring program at the Inkosi Albert Luthuli Central Hospital

You have been invited to participate in this research study.

You have been informed about the study by the researcher.

You may contact Ms Jessica Paken at the University of KwaZulu-Natal, Audiology Department, on 031-2607548 any time if you have any questions about the research.

You may contact the**:**
BIOMEDICAL RESEARCH ETHICS ADMINISTRATION
University of KwaZulu-Natal
Research Office, Westville Campus
Govan Mbeki Building
Private Bag X 54001, Durban, 4000
KwaZulu-Natal, SOUTH AFRICA
Tel: 27 31 2604769 - Fax: 27 31 2604609
Email: BREC@ukzn.ac.za, if you have any questions about your rights as a research participant.

Your participation in this research is voluntary, and you will not be penalized or lose benefits if you refuse to participate or decide to stop.

If you agree to participate, you will be given a signed copy of this document and the participant information sheet which is a written summary of the research.

The research study, including the above information, has been described to me. I understand what my involvement in the study means and I voluntarily agree to participate.

____________________ ________________
Signature of Participant Date

_______________________ _________________
Signature of Witness (Where applicable) Date
